# Food allergy in Venezuelan school children

**DOI:** 10.1186/2045-7022-3-S3-P36

**Published:** 2013-07-25

**Authors:** PA Franca, D Cifarelli, E Lopez, L Sarmiento, L Machado, G Maria

**Affiliations:** 1 Lab inmunopatologia, Instituto de Biomedicina UCV, Caracas, Bolivarian Republic of Venezuela

## Background

Food allergy (FA) is an important health problem in western societies; however, there are few data available from developing countries. Studies based on self-reported symptoms range from 1.4% to 33%. In Venezuela, allergic diseases epidemiological data are few and old. Local information mentioned a higher prevalence of asthma in the lower socioeconomic segments of the population.

## Methods

We evaluated a group of 1800 unselected school children (6-12 years old) from 10 randomly selected schools belonging from different areas of low socio economical levels, from Caracas City and other Venezuela States, using a validated modified Graffar’s socioeconomic questionnaire and also by allergic rhinitis, asthma and atopic dermatitis validated questionnaire after ARIA, GINA and Hannifin criteria. Food allergy induces symptoms were evaluated in a medical history and physical examination according to NIAD (Guidelines for the Diagnosis and Management of Food Allergy). Skin prick testing for different allergens plus specific IgE (by ELISA and multiple antigen blot assay), complete blood count (COULTER), and serial feces examination for ova and parasites were performed in all children. Pre and post bronchodilator FEV1 and PEF spirometric measurements values were obtained.

## Results

We showed a prevalence's of rhinitis (from 52,6 to 69.9%), asthma (from 22.4 to 56.6%), atopic dermatitis (from 5.5 to 13.6%) and food allergy (from 5 to 15%). Skin tests were positive to *Blo t* (from 14.65 to 33.68%) and *D.pt* (from 22.63 to 38.16%). Skin prick test to food allergens were positive to Milk (from 5.4 to 11%) egg (from 3.4 to 5,4%) Wheat (3.2-5.4%), tomato (2.7-4.8%), Soy milk (3.3-7.7%) Orange (3.4-14.5%) and Fish (3.3-15.8%).

## Conclusion

We report and confirm a high prevalence of allergic respiratory diseases and food allergy in Venezuelan children employing additionally to questionnaires, a clinical exam and an immune diagnostic evaluation methodology. Skin prick test results revealed that allergic sensitization food allergens were different according to consuming habits and more common in children from metropolitan zone than those from Margarita Island in where parasites allergens skin prick test positivity were most prevalent.

## Disclosure of interest

None declared.

